# Repair lower extremity vascular injury using a parallel connection for reconstruction of vessel graft with autogenous vein

**DOI:** 10.3389/fcvm.2026.1778934

**Published:** 2026-06-26

**Authors:** Jian-ping Liu, Qian Yao, Zhi-biao Le, Jun-qi Xiao, Rong Ye, Qing Duan, Jie Peng, Xunhong Duan

**Affiliations:** 1 Suzhou Medical College, Soochow University, Suzhou, China; 2 Department of Vascular Surgery, The First Affiliated Hospital, the First College of Clinical Medicine, Gannan Medical University, Ganzhou, China; 3 Department of Gastroenterology, The First Affiliated Hospital, the First College of Clinical Medicine, Gannan Medical University, Ganzhou, China

**Keywords:** autogenous vein, lower extremity, parallel connection, reconstruction, vascular injury, vessel graft

## Abstract

**Objective:**

Insufficient inner diameter of the autologous vein can negatively affect outcomes in vascular injury repair. This study reports the use of the parallel connection method to improve outcomes and assesses the efficacy and safety of reconstructive treatment for lower extremity vascular injuries using parallel connection vessel grafts from autogenous veins.

**Method:**

This retrospective study included 54 patients who underwent vascular reconstruction for lower extremity vascular injury between March 2015 and January 2023. Patients received either a parallel connection autogenous vein graft or direct autogenous vein reconstruction. Clinical and follow-up data were analyzed, and the two groups were compared in terms of thrombosis rate, amputation rate, and anastomosis time.

**Result:**

The parallel connection method proved more effective than direct autogenous vein grafting for both arterial and venous reconstruction. In arterial reconstruction, the parallel connection group had a significantly lower thrombosis rate (5.88% vs. 53.85%) and amputation rate (0% vs. 38.46%) compared with the direct autogenous vein graft group (both *P* < 0.05). For venous reconstruction, the thrombosis rate was also significantly lower in the parallel connection group than in the direct autogenous vein graft group (7.69% vs. 57.14%, *P* < 0.05). However, the procedure time was longer for the parallel connection method than for direct autogenous vein grafting (*P* < 0.05). No pseudoaneurysm, stenosis, or thromboembolism was observed in the target vessels during the follow-up period (23–60 months).

**Conclusion:**

The results of this study indicate that the parallel connection method yields lower rates of thrombosis and amputation compared with direct autogenous vein grafting in vascular injury reconstruction, although it requires longer operative time. This study has several limitations, including its retrospective design, small sample size, and potential selection bias. Therefore, larger prospective studies are needed to validate these findings.

## 1 Introduction

Peripheral vascular injuries represent a critical clinical entity frequently encountered in traumatic and surgical emergencies (1–4). Delayed intervention may precipitate limb amputation in severe cases and poses significant mortality risks (5–8). Autologous venous grafting remains the gold standard for vascular reconstruction owing to its biocompatibility and infection resistance (1, 9–12), with the great saphenous vein serving as the preferred conduit in extremity revascularization (13, 14). However, inherent limitations arise when donor vein caliber inadequately matches recipient vessel dimensions—a prevalent challenge in complex injuries. Inadequate luminal diameter of autologous veins frequently results in conduit–target vessel mismatch, generating excessive anastomotic tension that increases the risk of stenosis and thrombotic complications (15). While prosthetic grafts offer an alternative in select scenarios (1, 9), their clinical utility is constrained by limited availability in resource-restricted settings and contraindications in contaminated wounds owing to heightened infection risks (16). To address these challenges, we utilized the autologous saphenous vein with a parallel connection technique to construct a suitable vascular graft. This retrospective cohort study provides the first comprehensive analysis of the technical feasibility and clinical outcomes associated with parallel connection venous grafting in extremity vascular trauma.

## 2 Methods

### 2.1 Patient selection

**Figure 1:**
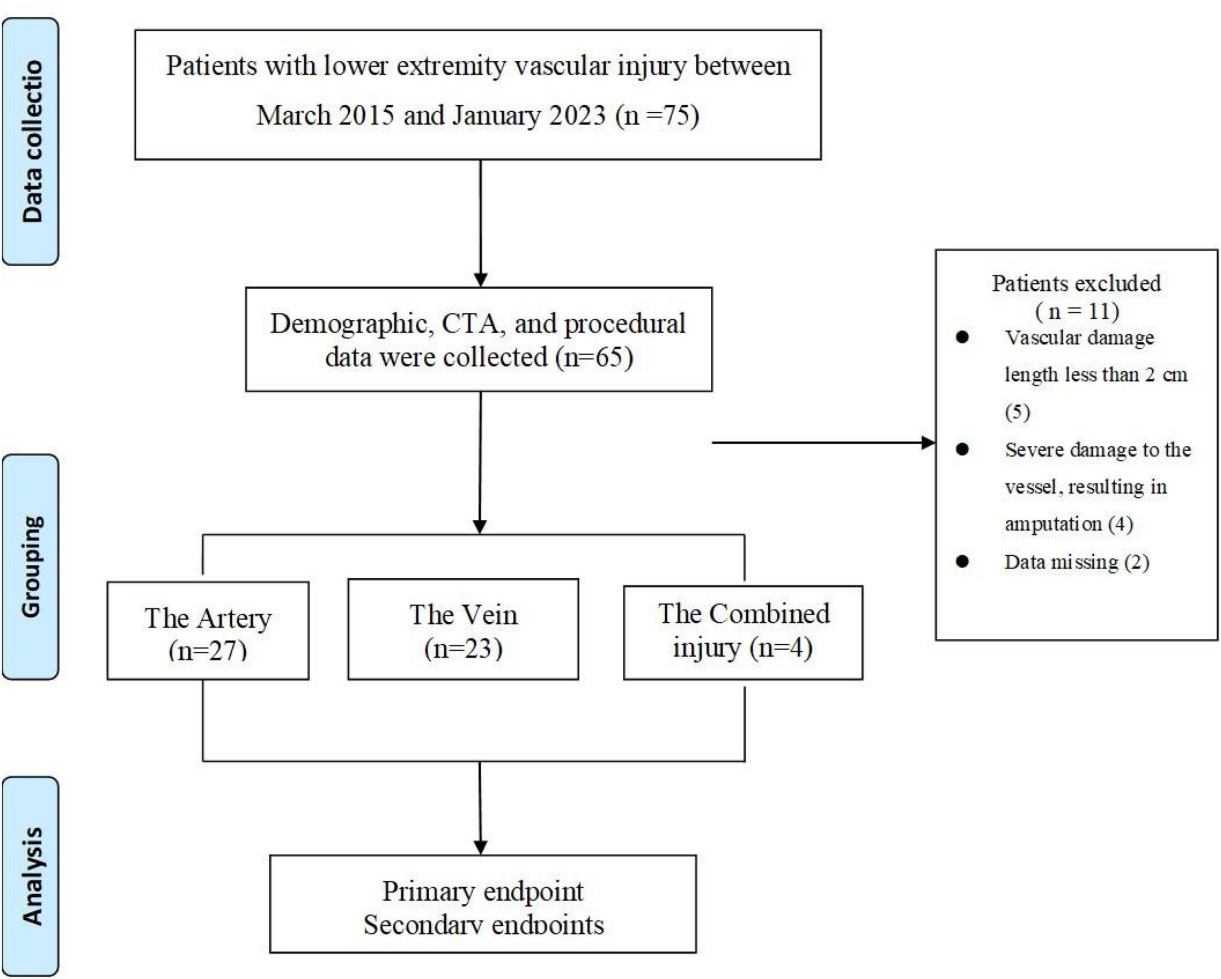
Flowchart of the present study.

We included cases treated at our center between March 2015 and January 2023 for the repair of lower extremity vascular injuries, using either direct autogenous vein grafts or parallel connection techniques. Inclusion criteria were as follows: (1) lower extremity vascular injury, (2) vascular damage length greater than 2 cm, and (3) the vessel graft harvested from an autogenous vein. Based on the graft vessel, patients were divided into the parallel connection group and the autogenous vein direct anastomosis group. The rate of thrombosis, amputation, and reconstruction time were compared between the two groups (Figure 1). Consent to utilize data was provided by both the patients and their families, and the study protocol was approved by the institutional ethics committee. The characteristics of the included patients and their baseline data are presented in Table 1.

**Table 1:** Gender and age of the 54 patients, and cause, type, and anatomical position of the vascular injury.

Variable	Artery (*n* = 27)	Vein (*n* = 23)	Combined injury (*n* = 4)
Gender (male), %	45 (83.3%)		
Age (year) (mean ± SD)	52.2 ± 8.7		
Trauma (*n*), %	15 (55.6)	14 (60.9)	2 (50)
Traffic accident (*n*), %	11 (40.7)	9 (39.1)	1 (25)
Iatrogenic (*n*), %	1 (3.7)		1 (25)
Blunt (*n*), %	6 (22.2)	6 (26.1)	1 (25)
Penetrating (*n*), %	21 (77.8)	17 (73.9)	3 (75)
Femoral vascular (*n*), %	6 (22.2)	11 (47.8)	1 (25)
Popliteal vascular (*n*), %	8 (29.6)	12 (52.2)	
Femoral-popliteal combined (*n*), %	13 (48.2)		4 (100)

### 2.2 Operating procedure

Autogenous vein grafts were harvested from the great saphenous vein on the ipsilateral side or the opposite side of the injury, or from the small saphenous vein on the same side. Venous branches were ligated to prevent postoperative leakage. During graft harvesting, the direction of the vein valve was marked to ensure correct orientation and antegrade flow after implantation. In patients undergoing direct autogenous vein grafting, the proximal and distal ends of the injured vessel were connected using an end-to-end anastomosis technique. In contrast, the parallel connection technique required the initial construction of a novel vascular conduit, which was then interposed between the proximal and distal ends of the target vessel and secured with end-to-end anastomoses to complete the vascular repair. In patient with combined arterial and venous injuries, the arteries were reconstructed using vascular prosthesis. The choice of surgical procedure was determined by the patient's clinical condition. For venous injuries, the “partial parallel connection” technique was used whenever possible to preserve valve function. For arterial injuries, the decision was guided by the duration of limb ischemia and the length of the vascular defect: In cases with prolonged limb ischemia (6–8 h) and a defect length exceeding 10 cm, “partial parallel connection” or direct anastomosis was preferred; in cases with limb ischemia lasting less than 6 hours, the “complete parallel connection” method was favored. Patients with no potential for limb salvage underwent direct amputation, while those with hemodynamic instability underwent direct ligation to control bleeding; such patients were excluded from this study. In this retrospective series, intraoperative diameter measurements were not systematically recorded for all patients. Technical success was defined as immediate intraoperative patency, confirmed by direct visual inspection and palpable distal pulse (for arteries) or refill (for veins).

### 2.3 Surgical technique: parallel connection methods

**Figure 2:**
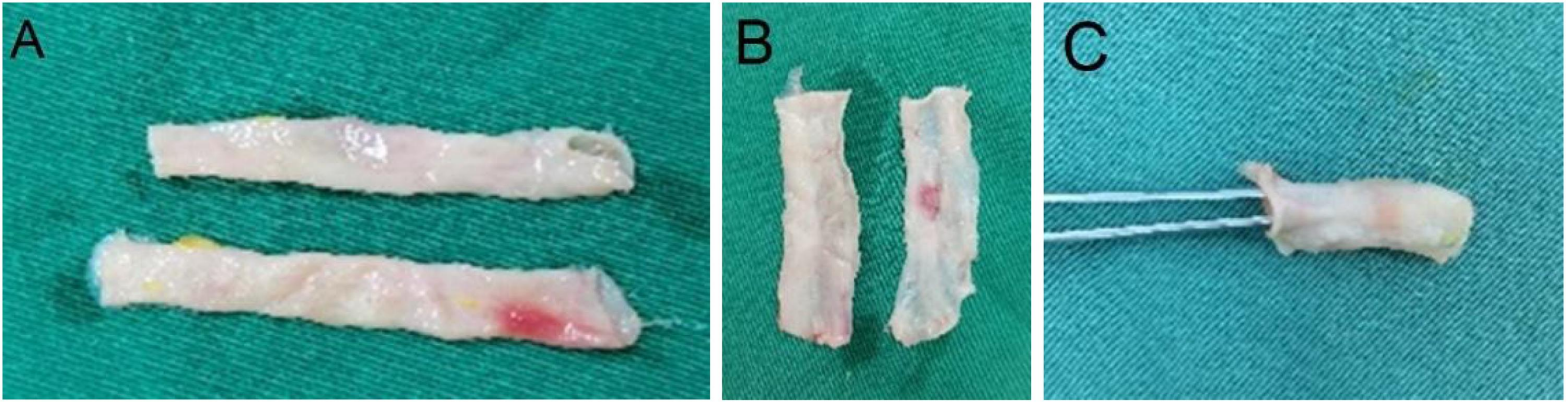
Complete parallel connection. **(A)** Two similar-length great saphenous veins; **(B)** longitudinal incision; and **(C)** graft vessel.

**Figure 3:**
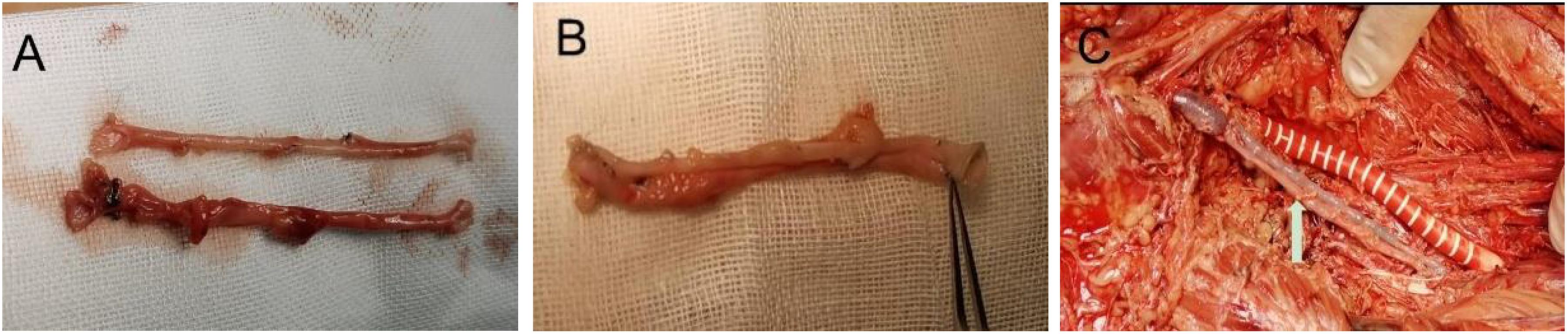
Partial parallel connection for graft vessel to repair the femoral-popliteal vein injury. **(A)** Two similar-length great saphenous veins; **(B)** end point longitudinal incision with partial parallel connection; and **(C)** repair of venous injury, with the graft vessel indicated by a white arrow.

**Figure 4:**
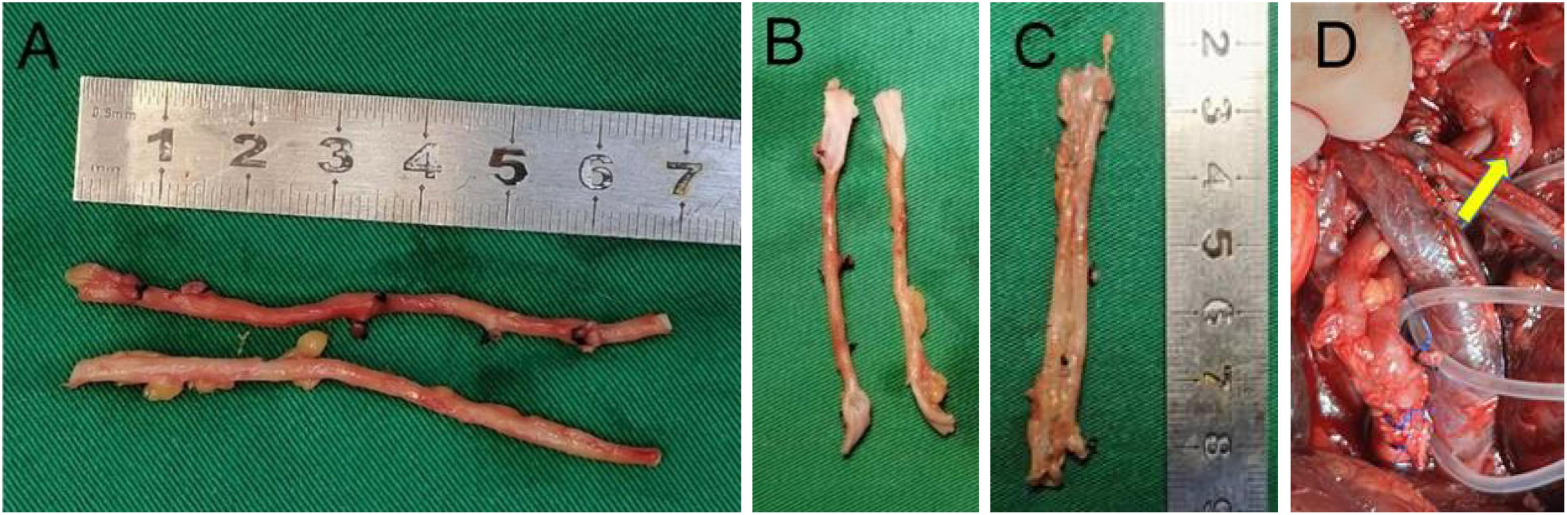
Partial parallel connection for graft vessel to repair the femoral-popliteal artery injury. **(A)** Two similar-length great saphenous veins; **(B)** end point longitudinal incision; **(C)** with the partial parallel connection; and **(D)** repair of arterial defect, with the graft vessel indicated by the yellow arrow.

We spliced autogenous veins into two types of vascular conduits designed to accommodate both the caliber and length of the injured vessel, which were designated as complete and partial parallel connections. *Complete parallel connection*: Both veins were incised longitudinally along their entire length and sutured side to side to create a uniformly enlarged conduit using 7-0 PROLENE line (Figure 2). *Partial parallel connection*: Only the two ends of each vein were incised longitudinally and sutured, leaving the middle portion intact. This preserved native valve function and was preferred for longer defects (Figures 3, 4). After connection, a new graft conduit was formed, and caliber enlargement was performed.

### 2.4 Anticoagulation and antiplatelet therapy

Systemic heparinization (100 U/kg) was administered to patients without bleeding risk; for high-bleeding-risk patients, local heparin flushing (25 IU/mL) was used. Postoperative: Venous injury alone: anticoagulation with low-molecular-weight heparin (100 IU/kg, q12h) for 5–7 days followed by warfarin (INR 2.0–2.5) or rivaroxaban (20 mg, QD) for 6 months. Arterial injury: aspirin 100 mg/day or clopidogrel 75 mg/day plus rivaroxaban (10 mg, QD) for at least 6 months. Patients at high risk for thrombosis received extended treatment according to individual circumstances. Early functional exercises were recommended to reduce the risk of thrombosis.

### 2.5 Antibiotics and laboratory tests

During hospitalization, muscle enzyme levels, coagulation function, and routine blood tests were closely monitored. All patients received prophylactic intravenous antibiotics (cefazolin 2 g or clindamycin 600 mg in case of penicillin allergy) within 30 min before skin incision, continued for 24–48 h postoperatively. In contaminated wounds, antibiotic course was extended based on wound culture results. No surgical site infection or graft infection was observed in either group.

### 2.6 Follow-up

Physical examination—including palpation of arterial pulses and clinical manifestations such as extremity edema—was performed, though data were not recorded for these assessments. Postoperative follow-up was conducted at 1, 3, 6, and 12 months, and annually thereafter. Duplex ultrasound was performed in nearly all patients. Coagulation analysis was also completed for patients who underwent venous reconstruction. For patients with arterial reconstruction, computed tomography angiography was performed at 12 months postoperatively or when clinically indicated.

### 2.7 Statistical analysis

Descriptive statistical analyses were performed to summarize the demographic and clinical characteristics of the patient cohort. Analysis of variance (ANOVA) was used for continuous variables, and chi-square or two-tailed Fisher's exact tests were applied for categorical variables. All statistical analyses were conducted using SPSS Statistics (version 22.0, IBM). A *P*-value < 0.05 was considered statistically significant.

## 3 Results

The study cohort comprised 45 (83.3%) men and 9 (16.7%) women, with a mean age of 52.2 ± 8.7 years. There were 21 (38.9%) cases of traffic accident, 31 (57.4%) cases of trauma, and 2 (3.7%) cases of iatrogenic injury. Penetrating injuries were observed in 41 (75.9%) patients, while 13 (24.1%) patients sustained blunt injuries. Twenty-seven (50%) patients presented with only arterial injury, 23 (42.6%) with only venous injury, and four with combined injuries. Among the 27 patients with only arterial injuries, there were six cases (22.2%) of femoral artery injury, eight (29.6%) cases of popliteal artery injury, and 13 (48.2%) cases of combined injury. Among 23 lower extremity venous injury cases, the injury was to the superficial femoral vein in 11 (47.8%) cases and the popliteal vein in 12 (52.2%) cases. All four (100%) patients with combined arterial and venous injuries sustained femoropopliteal vessel injuries. Parallel connection vessel grafting and autogenous vein grafting were performed for reconstruction of vascular damage. For arterial injuries, seven patients underwent complete parallel connection reconstruction and 10 underwent partial parallel connection reconstruction. For venous injuries, four patients underwent complete parallel connection reconstruction and nine underwent partial parallel connection reconstruction. Among the four patients with combined arterial and venous injuries, one underwent arterial reconstruction with a vascular prosthesis and venous repair using the parallel connection technique. In the remaining three patients, both the artery and vein were reconstructed using the parallel connection technique. Direct autologous vein grafting was performed in 13 arterial cases (43.3%, 13/30) and 14 venous cases (51.9%, 14/27). The duration of target vascular blood flow restoration (from target vessel dissection to clamp release) was 3.1 ± 0.3 h. Technical success rate was 100%. Regardless of whether arterial or venous reconstruction was performed, the effectiveness of the parallel connection method was higher than that of autogenous vein direct grafting. For arterial reconstruction, the parallel connection group demonstrated significantly lower rates of thrombosis [5.88% [0.1%–28.7%] vs. 53.85% [95% CI: 25.1%–80.8%], *P* < 0.05] and amputation [0% [95% CI 0–20.6%] vs. 38.46% [95% CI 24.5–91.5%], *P* < 0.05] compared with the direct anastomosis group. All amputations were major amputations (above ankle). No minor amputations (toe or transmetatarsal) occurred. For venous reconstruction, the parallel connection group similarly showed a significantly lower thrombosis rate [7.69% [95% CI: 0.20%–36.03%] vs. 57.14 [95% CI: 28.90%–82.41%], *P* < 0.05] than the direct anastomosis group. For arterial reconstruction, the mean anastomosis time was 48.0 ± 4.3 min for the parallel connection method versus 32.7 ± 2.1 min for direct anastomosis (*p* = 0.005). For venous reconstruction, the corresponding times were 51.5 ± 5.3 min and 31.1 ± 2.2 min, respectively (*p* = 0.002) (Table 2). Baseline characteristics were compared between the direct autogenous vein reconstruction group and the parallel connection group (Table 3). Early thromboembolism occurred in one case of both arterial and venous injury, and stable patency was obtained after percutaneous mechanical clearance. No false aneurysm, stenosis, or thromboembolism occurred in the target vessels during follow-up period of 23–60 months.

**Table 2:** Thromboembolism, amputation rate, and time to vascular anastomosis of the two vascular reconstruction methods.

Method	Thromboembolism		Amputation	Anastomosis time	
	Artery (*n* = 30)^a^	Vein (*n* = 27)^b^	Artery (*n* = 30)^a^	Artery (*n* = 30)^a^	Vein (*n* = 27)^b^
Parallel connection (%)	1	1	0	48 ± 4.3	51.5 ± 5.3
Direct anastomosis (%)	7	8	5	32.7 ± 2.1	31.1 ± 2.2
*P*	0.009	0.000	0.009	0.005	0.002

a Thirty arteries received autogenous vein repair.

b Twenty-seven veins received autogenous vein repair; amputation defined as major amputation (below knee or above knee). No minor amputations were required in this series.

**Table 3:** Baseline characteristics were compared between the direct autogenous vein reconstruction group and the parallel connection group.

Method	Age	Gender		Hypertension	Diabetes	Smoking
		Male	Female			
Parallel connection	52.0 ± 9.4	24	6	7	3	16
Direct anastomosis	52.3 ± 7.9	23	4	5	3	13
*P*	0.204	0.734		0.751	0.613	0.793

## 4 Discussion

Peripheral vascular injury is a frequent cause of amputation and death, occurring both among civilians and on the battlefield (5–8). Penetrating and blunt trauma are common types of vascular injury (4), while iatrogenic injury has become increasingly recognized in clinical interventional operations. Blunt trauma is the most common type of vascular injury among civilians, and penetrating trauma the most frequently encountered on the battlefield (5, 17, 18). Lower extremity vessel injury, involving the femoral and popliteal arteries, is the most common peripheral vascular injury (17). As long as the condition permits, it is necessary to repair the vascular, which has been formed a consensus. Timely diagnosis and surgical intervention to stop bleeding and restore blood flow remain the primary goals (1, 17, 19). In the past, vascular ligation was the standard life-saving treatment for vascular injury (20); however, vascular repair has been shown to reduce amputation rates, in both military and civilian contexts (1, 17–21). For vascular injuries, direct suture, patch repair, and end-to-end anastomosis are the most common surgical procedures (6). For vascular defects longer than 2 cm, direct anastomosis is often unsuitable due to excessive tension at the anastomotic site, and grafting is recommended. (9, 20, 22, 23).

Although endovascular treatment provides great benefit in the management of vascular disease, open surgery remains the golden standard for vascular repair (1, 10, 12). Vascular prostheses and autologous veins are the primary replacement sources for human vascular defects. Vascular prostheses are associated with potential risks such as infection, haemorrhage, leakage, and thrombosis. Compared with autologous veins, vascular prostheses are not recommended for use in cases of wound or other potential infection (9), particularly in arterial injury, due to the risk of serious complications that may be life-threatening. Vascular prostheses cannot be routinely used in grassroots hospitals in developing countries. Temporary intravascular shunts can shorten the duration of ischemia while patients are being transferred to higher-level hospitals (24). However, patient recovery may be optimal if treatment can be managed at a local hospital (6). In addition, vascular prostheses have the disadvantages of being expensive and exhibiting poor histocompatibility. Compared with vascular prostheses, autologous veins offer several advantages: (1) stronger anti-infection ability, suitable even in infected wounds (9); (2) good histocompatibility, promoting rapid healing; (3) reduced occurrence of haemorrhage and leakage; (4) lower rates of thrombosis and thromboembolism and higher rates of patency (12); and (5) lower cost. The disadvantage is that the autogenous vein without a matching caliber for the target vascular, at the most time, which causes anastomosis stoma tension.

Previously, vascular anastomoses were trimmed into oblique shapes to reduce difficulties. However, in cases of significant differences in internal diameters, anastomosis is difficult to accomplish. If there is a significant mismatch in caliber, forcing anastomosis with the autogenous vein would result in anastomosis stoma stenosis and increase the risk of thromboembolism. The worst outcome is vascular obstruction. To reduce the incidence of this adverse outcome, we constructed graft conduits and minimized the gap in diameter with the target vessel. We called this method the “parallel connection” method. This method can be divided into two categories: (1) complete parallel connection and (2) partial parallel connection. The former procedure requires more time to construct the donor graft vessel. This graft vessel has a uniform enlarged lumen, which is especially suitable for shorter vascular defects. The latter procedure is easier and requires less time for graft vessel construction. With complete parallel connection, it is possible to construct an entire enlarged conduit, although normal valve function cannot be achieved. Compared with complete parallel connection, partial parallel connection can maintain original valve function of the autogenous vein, which is crucial for venous blood flow. The technique developed here represents an effective solution to the problem of inadequate autogenous grafting diameter. Compared with direct autogenous vein anastomosis, the parallel connection method yields lower rates of thrombosis, thromboembolism, and amputation. However, the parallel connection method is more time-consuming than direct anastomosis.

The most commonly involved vessel in arterial injuries of the extremities is the superficial femoral artery (25, 26). There is no disputing the fact that vascular repair has led to a significant reduction in amputation rates after vascular injury (27). Restoration and preservation of vascular flow are important factors for limb salvage after peripheral vascular injury (28). Tension-free repair is recommended as the primary choice for arterial injuries with low-velocity penetrating wounds (27). Vessel grafts should be taken into consideration in cases where the length of vascular damage rules out the possibility of primary repair. Although graft material remains controversial, autogenous vein is still the first choice. In our study, where possible, we used autogenous vessels rather than vascular prostheses due to infection risks and difficult timely access to artificial vessels. Our research shows that autogenous veins have advantages such as lower thromboembolism rates (29) and higher resistance to infection (30). To shorten the operation time and restore blood flow as soon as possible, the partial parallel connection was the first choice for reconstruction in the case of vascular injury, particularly long artery defects. Despite the partial parallel connection graft consisting of two independent lower-caliber vessels, flux is satisfactory due to high blood pressure and flow velocity of blood. To maintain unimpeded blood flow, the orientation of the venous valves in the graft must be aligned with the direction of blood flow. Compared with direct autogenous vein anastomosis, the parallel connection technique has a higher limb salvage rate and lower thrombosis rate. In addition, the result showed that the amputation rate was lower in patients who underwent parallel connection graft vessel repair compared with those who underwent direct anastomosis. It must be acknowledged that unsatisfactory outcomes with direct autogenous vein or vascular prosthesis repair may reflect our inexperience during the early stage of this study. Importantly, the material used to repair blood vessels is not the only factor in the decision to amputate. Fewer postoperative complications also contribute to limb salvage. If time permits, partial parallel connection is preferred for reconstruction of arterial injuries that cannot be directly repaired or anastomosed.

The repair of peripheral venous injury is controversial. Critics have major concerns that venous repair will result in venous thrombosis and subsequent pulmonary embolism (31, 32). Some studies report that venous ligation does not increase the risk of mortality and amputation (33, 34). Other studies have shown that venous repair carries a low risk of pulmonary embolism and deep venous thrombosis, and the risks between repair ligation are equivalent (35, 36). The popliteal vein, femoral vein, and iliac vein are recommended for repair (5, 36, 37), and procedures involving these veins are beneficial for reducing arterial blood flow resistance, reducing tissue edema, and improving limb salvage rate (37). Vein repair is critical in young patients (36). We believe that the clinical consequences of major vein ligation resemble the complications of lower-extremity deep vein post-thrombotic syndrome (PTS). Although collateral circulation is abundant, it cannot fully compensate for the venous hypertension caused by venous obstruction, which may lead to varicose veins, skin pigmentation, venous ulcers, and a severely impaired quality of life (38). Young patients, due to their higher levels of exercise compared with older people, are more likely to have more serious complications resulting from major vein obstructions. In cases of combined venous and artery injuries, trauma and ischemic reperfusion result in severe tissue edema (1, 39). Therefore, increasing venous reflux is key to reducing limb edema (31, 39). It is essential to reconstruct damaged major veins whenever the patient's condition permits, particularly in younger patients. In this study, for venous injuries that were not amenable to direct anastomosis, we performed repair using either direct autogenous vein grafting or the parallel connection technique, rather than bypass surgery. Ideally, uniformly enlarged graft vessels could be constructed using the parallel connection method at any time. However, owing to time constraints, we sometimes had to use the partial parallel connection technique for longer venous defects. Due to time constraints, we repaired longer venous defects with the partial parallel connection method in some cases. Our study shows that parallel graft vessels can lower the probably of obstruction compared with direct autogenous vein anastomosis.

Other treatments during the perioperative period are also equally important. In order to prevent thrombosis and thromboembolism, postoperative anticoagulation or antiplatelet therapy, of the appropriate dose and at the right time, is crucial. Unlike arteries, veins have slow blood flow and ongoing repair processes, both of which are high-risk factors for thrombosis. We recommend that long-term pressure treatment be provided after vein repair to effectively prevent lower limb venous thrombosis.

Ultimately, whether or not the limb can be saved following vascular trauma depends upon many factors: the anatomical location of the vascular injury; the duration of ischemia; and the extent of tissue, nerve, and bone damage. Early diagnosis, bleeding control, restoration of blood flow, and reduction of ischemia-reperfusion injury time are crucial in saving the limb. The involvement of specialized vascular surgeons significantly improves prognosis. However, endovascular treatment cannot replace open surgery, which remains the most durable and reliable method, especially in young patients with extensive vascular defects. Stenting carries the risk of intimal hyperplasia and in-stent reocclusion, which are concerning, especially in young patients. Other techniques to address caliber mismatch include spatulated anastomosis, Linton patch angioplasty, or creation of an arteriovenous fistula. These were not employed in our series as we focused on the parallel connection method. Future comparative studies would be valuable to determine the optimal approach.

## 5 Conclusion

The findings of this study indicate that the parallel connection method for vascular injury reconstruction yields lower rates of thrombosis and amputation than direct autogenous vein grafting, despite longer procedure time. The limited sample size in this study underscores the need for larger prospective investigations.

### 5.1 Clinical perspectives

- Vascular injury to the lower extremities is a common surgical emergency where improper management can lead to amputation or be life-threatening. The great saphenous vein is the most frequently used source of autologous grafts for repair; however, its application is often limited by insufficient caliber.
- Using the great saphenous vein in a parallel connection configuration effectively increases the graft's internal diameter and yields favorable clinical outcomes.
- In scenarios where prosthetic grafts are unavailable or contraindicated, the application of this technique can reduce the amputation rate.

## 6 Limitations

Several limitations of this study should be acknowledged. First, the retrospective, non-randomized design inherently carries the risk of selection bias, as the choice of surgical technique was not allocated prospectively. Second, the relatively small sample size and single-center setting limit the statistical power and generalizability of our findings. Third, although all procedures were performed or directly supervised by senior surgeons with more than 10 years of vascular surgical experience, a learning curve associated with the parallel connection technique may still exist, which could influence the reproducibility of our results in other centers. Fourth, some data could not be retrieved retrospectively, which constitutes an inherent limitation. The small sample size and retrospective design, along with the fact that data were not recorded at strictly defined time points and follow-up visits were not always performed on schedule, precluded a standardized reporting format. Therefore, larger, prospective, multicenter studies are needed to validate our observations.

## Data Availability

The raw data supporting the conclusions of this article will be made available by the authors, without undue reservation.
